# The most used and most helpful facilitators for patient-centered medical home implementation

**DOI:** 10.1186/s13012-015-0246-9

**Published:** 2015-04-19

**Authors:** Randall C Gale, Steven M Asch, Thomas Taylor, Karin M Nelson, Jeff Luck, Lisa S Meredith, Christian D Helfrich

**Affiliations:** Center for Innovation to Implementation (Ci2i), VA Palo Alto Health Care System, 790 Willow Road, Menlo Park, CA 94025 USA; Division of General Medical Disciplines, Stanford University, Palo Alto, CA USA; US Department of Veterans Affairs, Seattle-Denver Center of Innovation for Veteran-Centered and Value-Driven Care, Seattle, WA USA; Department of Medicine, University of Washington School of Medicine, Seattle, WA USA; Department of Health Services, University of Washington School of Public Health, Seattle, WA USA; College of Public Health and Human Sciences, Oregon State University, Corvallis, OR USA; VA HSR&D Center for the Study of Healthcare Provider Behavior, Sepulveda, CA USA; RAND Corporation, Santa Monica, CA USA

**Keywords:** Patient-centered medical home, Implementation resources, Provider self-efficacy

## Abstract

**Background:**

Like other transformative healthcare initiatives, patient-centered medical home (PCMH) implementation requires substantial investments of time and resources. Even though PCMH and PCMH-like models are being implemented by multiple provider practices and health systems, little is known about what facilitates their implementation. The purpose of this study was to assess which PCMH-implementation resources are most widely used, by whom, and which resources primary care personnel find most helpful.

**Methods:**

This study is an analysis of data from a cross-sectional survey of primary care personnel in the Veterans Health Administration in 2012, in which respondents were asked to rate whether they were aware of and accessed PCMH-implementation resources, and to rate their helpfulness. Logistic regression was used to produce odds ratios for the outcomes (1) resource use and (2) resource helpfulness. Respondents were nested within clinics, nested, in turn, within 135 parent hospitals.

**Results:**

Teamlet huddles were the most widely accessed (80.4% accessed) and most helpful (90.4% rated helpful) resource; quality-improvement methods to conduct small tests of change were the least frequently accessed (42.4% accessed) resource though two-thirds (66.7%) of users reported as helpful. Supervisors were significantly more likely (ORs, 1.46 to 1.86) to use resources than non-supervisors but were less likely to rate the majority (8 out of 10) of resources as “somewhat/very helpful” than non-supervisors (ORs, 0.72 to 0.84). Longer-tenured employees tended to rate resources as more helpful.

**Conclusions:**

These findings are the first in the PCMH literature that we are aware of that systematically assesses primary care staff’s access to and the helpfulness of PCMH implementation resources. Supervisors generally reported greater access to resources, relative to non-supervisors, but rated resources as less helpful, suggesting that information about them may not have been optimally disseminated. Knowing what resources primary care staff use and find helpful can inform administrators’ and policymakers’ investments in PCMH-implementation resources. The implications of our model extend beyond just PCMH implementation but also to considerations when providing implementation resources for other complex quality-improvement initiatives.

**Electronic supplementary material:**

The online version of this article (doi:10.1186/s13012-015-0246-9) contains supplementary material, which is available to authorized users.

## Background

The patient-centered medical home (PCMH) is a model that emphasizes team-based, integrated care [1-3] and has been heralded as a potential solution to cost, quality, and access issues plaguing the US health care system [4-6]. PCMH models are generally characterized by: (a) the provision of comprehensive care by an integrated team of providers (either under a single roof or virtual) responsible for the majority of patients’ clinical needs; (b) a patient-centered philosophy that treats patients and family members as partners; (c) care that is coordinated across the continuum and between settings, that is accessible, and aligned with patient preferences; and (d) a demonstrated commitment to and engagement in quality-improvement (QI) activities [7]. Both private and government entities have invested substantially in PCMH implementation [8-10].

To successfully implement and sustain complex, evidence-based quality-improvement initiatives, organizations must be willing to invest the necessary time and resources [11,12]. Even when such investments are made, efforts may fail or dissemination may occur slower than desired [13]. Like other quality-improvement initiatives, PCMH implementation appears to require substantial assistance and supporting resources [14]. These resources might include policy guidance documents, in-person or virtual learning collaboratives, collections of “on-demand” resources (i.e., “toolkits”), brief daily meetings, registries, and systems for measuring the effectiveness of quality-improvement interventions [15]. The usefulness of a particular resource may differ across PCMH roles, for example, providers versus nurses or care managers versus dietitians. Although some research has evaluated the use of specific online quality-improvement resources [16], it remains unclear which implementation resources are most widely used, by whom, and which of those resources PCMH personnel find most useful. The scarcity of published research on the factors associated with uptake and spread is not unique to the PCMH setting [11].

The Veterans Health Administration (VHA) launched a nationwide PCMH initiative, called the Patient Aligned Care Teams (PACT), in 2010 [17] investing $822 million [9] in additional staff and instituting a nationwide training program and regional learning collaboratives [17]. In implementing PACT across hundreds of medical centers and clinics, VHA leaders aimed to strike a balance between standardization of certain core elements among facilities and opportunities to experiment and implement local solutions [17]. To support implementation, the VHA identified a core group of geographically disparate providers to implement structural changes and test strategies to bolster quality, efficiency, and patient satisfaction. Members of this group served as coaches for other local staff [17]. Five regional demonstration laboratories were established to assess the model’s impact and test methods of improvement with the goal of spreading successful ideas [17]. Although practice facilitation was not a formalized component of the VHA’s implementation strategy, in some cases, regional VHA networks provided internal coaches for PACT teams [18].

The VHA identified an extensive list of PACT-related training needs [19] and made available a wide variety of activities and resources to support PACT implementation. Table 1 lists each of the activities and resources, provides a statement of purpose or goal, and identifies an example for each. These activities and resources were generally intended to diffuse information about PACT, help define roles and responsibilities for PACT teams, improve communication, assist in goal setting, measurement, and process improvement activities, and ensure that PACT teams were able to track patients as they transitioned between care settings (e.g., inpatient to ambulatory). While this was the intended implementation strategy, the actual training and support likely varied; in particular, promised staffing increases to support the new model appear to have fallen short in many instances [19,20].

**Table 1:** **Description of PACT-related activities or resources to support implementation**

**Activity or resource**	**Purpose or goal**	**Example**
Local (e.g., work station or parent-facility) education sessions specifically about PACT	Introduce staff to the PACT model, team-based care and clarify expectations	A facility or team level in-service to provide an overview of PACT, define the roles and responsibilities of different team members, and to review metrics for measuring implementation progress
Regional or national learning collaboratives about PACT	Facilitate a common understanding of PACT, identify goals, and share experiences across the VHA	There were a series of six regional learning collaboratives that required all medical centers to send teams to attend. Collaboratives occurred quarterly, and each covered a different topic. The attending teams were expected to take lessons back to their facilities and share them with others
Measurement tools associated with PACT to help assess the team’s performance	Use of patient data to evaluate improvement benchmarks	Formative evaluation and feedback
Teamlet huddles	Improve communication among team members to better coordinate clinical care during a given tour of duty	Brief daily meetings before clinic, or during the day, to establish a game plan for the day, clarify primary areas of responsibility, and to identify any anticipated challenges
Regular teamlet meetings (other than huddles) to discuss process/performance improvement	Identify areas where performance is on track and areas where additional attention is needed	Formal weekly or monthly meetings to track and trend performance-improvement activities and discuss strategies for addressing deficiencies. This may be a stand-alone meeting or a designated portion of an existing standing meeting
Information systems to provide timely data and feedback to staff on PACT activities	Leverage use information technology so staff can make “real time” adjustments to processes in response to their performance	The Primary Care Management Module is a suite of software tools that can be used by primary care and other teams to assign patients to teams, assign patients to practitioners, and generate reports
New approaches to scheduling	Improve same day access to providers by allotting appointments for emergent issues	Proactively reviewing upcoming clinic schedules to convert appointments to telephone encounters or mid-level practitioner appointments, when appropriate
Quality-improvement methods to conduct small tests of change	Use established methods for implementing change, studying the impact of change, and making modifications to maximize results	Plan-Do-Study-Act (PDSA) cycles for implementing process improvement in the PACT team
Disease registries	Use existing or create new disease registries to evaluate care processes and outcomes, including disparities and to follow patients throughout the care trajectory	Diabetes, hypertension, and congestive heart failure registries
Online toolkit of care delivery and organization tools	Facilitate the peer-to-peer exchange of team- or facility-level quality-improvement tools or innovations	Online compendia of quality-improvement tools developed and implemented by at least one facility, that have been vetted by a group of peers, and that address at least one priority area of PACT implementation

We analyzed data from a national VHA survey to address the following research questions: Which of these activities and resources were most used and most helpful? How did assessments vary across different primary care team members? Our findings may help other health systems in thinking about how to prioritize investments in the development and dissemination of implementation-boosting resources.

## Methods

### Survey administration

We analyzed data from a nationwide, cross-sectional survey conducted in the VHA during the summer of 2012. Survey administration is described elsewhere [21]. Primary Care and Nursing clinical leadership were asked to distribute the web-based survey to all the staff working in primary care, regardless of whether or not they were a member of a designated PACT teamlet (a teamlet is comprised of a primary care provider, nurse care manager, nursing assistant or licensed nurse practitioner, and administrative associate) [22]. Surveys were anonymous and voluntary. The survey was disseminated to the staff in 20 of 21 VHA regional networks. Sites participating in two of the five regional demonstration laboratories were not surveyed because they fielded similar, but non-anonymous surveys not reported here. Facility-level identifiers were used to link survey data to VHA facility complexity scores, which is an administrative measure used to categorize facilities based on patient volume, scope of clinical services, teaching, and research activities [23].

Respondents were asked to rate the helpfulness of ten different PACT-related activities or resources (i.e., facilitators) encompassing PACT education and training, formal and informal team meetings, quality-improvement methods, measurement tools and data on performance, new models for scheduling, disease registries, and an online quality-improvement toolkit (Table 1). The facilitator items were developed in conjunction with the five regional demonstration laboratories based on early experiences with PACT and were selected to represent activities or resources that were widely applicable and thought to be important to the model. The survey instrument provided only the names of the activities and resources and not the intended goals or purposes nor examples of what their application looked like in the VHA.

### Outcomes

The first outcome of interest was whether respondents used a resource or participated in a PACT-related activity. Respondents who rated a resource or activity as “not helpful”, “somewhat helpful”, or “very helpful” were considered to have used or participated in it. Those who responded “not applicable”, “not involved”, or “don’t know” were categorized as non-users/participants. The second outcome was how helpful respondents rated each resource or activity (i.e., its utility). Responses were collapsed into two categories—“not helpful” and “somewhat/very helpful”—, and the sample was restricted to respondents who were categorized as having used or participated in each activity or resource.

### Data analysis

To account for nesting and the fact that helpfulness was contingent on resource use, a 3-level random intercept (survey respondents within 678 clinics, within 135 parent VHA health care systems/medical centers) two-part mixed model was estimated for each of the ten resources. The first equation in the two-part model predicted likelihood of use/participation (ten models) while the second part of the two-part model predicted the utility (helpfulness) of those resources (ten models).

Respondent-level covariates included: primary care role (e.g., provider, nurse, technician, etc.); years working in primary care; years working in the VHA; ethnicity; age; sex; having supervisory responsibilities (no supervisory responsibilities versus any, including team leads, first line supervisors, managers, and executives); percent of time spent working in primary care; and PACT teamlet membership. We also included a facility-level covariate for facility complexity, an index of the sophistication, and breadth of care provided at a given VHA medical center [23]. Odds ratios (ORs) and 95% confidence intervals (CIs) are reported. We used listwise deletion for infrequent covariate missingness.

Data analysis was conducted with Microsoft Excel (Redmond, WA, USA), SPSS version 21 (SPSS IBM, New York, USA), and R version 3.1 (R Foundation for Statistical Computing, Vienna, Austria). This study was determined to be a non-human subject research project by the Stanford University Institutional Review Board.

## Key results

### Survey respondent demographics

There were 6,464 survey respondents. With 13,742 support staff and more than 4,500 providers working in primary care at the end of 2011 [22], survey respondents accounted for approximately 35% of those working in VHA primary care. Majorities of respondents were female (71%), aged 40–59 years (59.9%), and non-Hispanic white (67.9%) (Table 2). A plurality (43.8%) had worked in the VHA for two to ten years, and the majority (84.2%) spent more than 80% of their time working in primary care. Most respondents (59.7%) reported no supervisory responsibilities. Primary care providers, which included physicians, nurse practitioners (NP), and physician assistants comprised 27.4% of the respondents; 17.6% were nurse care managers, which included registered nurses (RNs) and a small number of NPs; 17.3% were licensed practical nurses (LPNs), licensed vocational nurses (LVNs), or certified nursing assistants (CNAs); 11.3% worked in an administrative role; and 4% were medical/health technicians. The majority of respondents (79.3%) reported being assigned to a PACT teamlet.

**Table 2:** **Respondent characteristics,** ***n*** **= 6,464 survey respondents**

**Variable**	**Number**	**Percent**
Sex		
Male	1537	23.8
Female	4591	71.0
Unknown	336	5.2
Age (years)		
<20	13	0.2
20–29	277	4.3
30–39	1110	17.2
40–49	1707	26.4
50–59	2165	33.5
≥60	736	11.4
Unknown	456	7.1
Race		
White	4389	67.9
Black or African-American	606	9.4
American-Indian or Alaska Native	47	0.7
Asian	394	6.1
Native Hawaiian or other Pacific Islander	53	0.8
Other/Unknown	975	15.1
Hispanic ethnicity		
Yes	427	6.6
No	5374	83.1
Unknown	663	10.3
Time working in the VHA		
<6 months	162	2.5
6 months to 1 year	279	4.3
1–2 years	609	9.4
2–5 years	1504	23.3
5–10 years	1324	20.5
10–15 years	880	13.6
15–20 years	525	8.1
>20 years	805	12.5
Unknown	376	5.8
Percent time spent working in primary care		
<20	351	5.4
20–40	166	2.6
41–60	219	3.4
61–80	285	4.4
>80	5443	84.2
Primary job function in primary care		
Administrative	732	11.3
Dietician	88	1.4
LPN/LVN/CNA	1119	17.3
Mental health professional	110	1.7
Nurse care manager	1136	17.6
Nurse case manager	246	3.8
Other or unknown	100	1.5
Other RN	354	5.5
Pharmacist	375	5.8
Provider	1769	27.4
Social worker	178	2.8
Technician	257	4.0
PACT teamlet membership		
Yes	5129	79.3
No/not applicable to role in the VHA	1198	18.5
Not sure	137	2.1
Supervisory responsibility		
None	3858	59.7
Team leader	1735	26.8
Higher than team leader	545	8.5
Unknown	326	5.0

### Use and utility of PACT implementation resources

The most widely used resources were teamlet huddles (80.4% of respondents), local education sessions about PACT (75.7%), measurement tools to assess team performance (74.7%), and regular teamlet meetings to discuss improvement (72.5%) (Table 3). Quality-improvement methods to conduct small tests of change were the least used by 42.4% of respondents.

**Table 3:** **Utility of PACT implementation activities or resources,** ***n*** **= 6,464 survey respondents**

**Activity or resource**	**Not helpful**	**Somewhat or very helpful**	**Don’t know/not involved**
Local (e.g., work station or parent-facility) education sessions specifically about PACT.	1146 (17.7)	3752 (58.0)	1566 (24.2)
Regional or national learning collaboratives about PACT	1102 (17.1)	3175 (49.1)	2187 (33.9)
Measurement tools associated with PCMH to help assess your team’s performance	1371 (21.2)	3460 (53.5)	1633 (25.3)
Teamlet huddles	500 (7.7)	4694 (72.6)	1270 (19.6)
Regular teamlet meetings (other than huddles) to discuss process/performance improvement	546 (8.5)	4134 (64.0)	1784 (27.6)
Information systems to provide timely data and feedback to staff on PACT activities	1052 (16.3)	3430 (53.1)	1982 (30.7)
New approaches to scheduling	1182 (18.3)	3077 (47.6)	2205 (34.1)
Quality-improvement methods to conduct small tests of change	913 (14.1)	1830 (28.3)	3721 (57.6)
Disease registries	724 (11.2)	3092 (47.8)	2648 (40.9)
Online toolkit of care delivery and organization tools	913 (14.1)	2134 (33.0)	3417 (52.9)

The resources most often rated somewhat or very helpful were “teamlet huddles” (by 90.4% of the respondents who used that resource), “regular teamlet meetings to discuss improvement” (88.3%), “disease registries” (81%), and “local education sessions about PACT” (76.6%). Although less often reported as helpful, two-thirds (66.7%) of those who conducted small tests of change rated as helpful.

### Significant predictors of resource use

Accounting for all other covariates, supervisors were significantly more likely (OR range, 1.46 to 1.86) to report the use of each of the ten resources than non-supervisors (Table 4). Non-PACT team members were significantly less likely (OR range, 0.17 to 0.78) to report the use of each resource than PACT team members. Longer tenure in the VHA was positively associated with use of seven out of ten resources.

**Table 4:** **Significant odds ratios (95% confidence intervals) for predictors of resource use** ^**1**^

**Covariate**	**Local PACT education**	**PACT collaborative**	**Measures**	**Teamlet huddles**	**Teamlet meetings**
Supervisor (versus not)	1.68 (1.43–1.97)	1.55 (1.35–1.79)	1.86 (1.58–2.18)	1.63 (1.36–1.95)	1.63 (1.39–1.92)
Time worked in VHA (ref. is <0.5 years)					
0.5–1 years					
1–2 years	1.86 (1.26–2.75)		1.95 (1.3–2.94)		
2–5 years	2.08 (1.43–3)	1.58 (1.12–2.25)	2.14 (1.48–3.1)		
5–10 years	1.77 (1.22–2.56)	1.57 (1.11–2.23)	1.93 (1.34–2.8)		
10–15 years	2.08 (1.4–3.06)	1.67 (1.15–2.41)	1.75 (1.19–2.59)		
15–20 years	2.03 (1.35–3.06)	1.68 (1.14–2.48)	1.92 (1.25–2.94)		
>20 years	2.64 (1.75–3.97)	1.68 (1.16–2.44)	2.39 (1.58–3.6)		
Role in primary care (ref. is provider)					
Administrative	0.77 (0.61–0.98)			0.7 (0.54–0.92)	1.28 (1.01–1.63)
Dietician	0.54 (0.32–0.92)		0.43 (0.25–0.73)	0.15 (0.09–0.27)	0.52 (0.3–0.9)
LPN/LVN/CNA	1.31 (1.05–1.63)	1.8 (1.48–2.2)	1.48 (1.16–1.88)		2.12 (1.67–2.69)
Mental health professional			0.39 (0.25–0.62)	0.46 (0.28–0.76)	
Nurse care Manager	1.3 (1.04–1.62)	1.42 (1.16–1.73)	1.52 (1.2–1.93)	2.14 (1.57–2.92)	1.86 (1.49–2.32)
Nurse case manager					
Other				0.39 (0.23–0.65)	
Other RN		1.34 (1.02–1.75)			1.93 (1.42–2.64)
Pharmacist			0.57 (0.42–0.78)	0.19 (0.14–0.27)	
Social worker			0.44 (0.31–0.64)	0.38 (0.25–0.58)	
Technician			0.68 (0.49–0.95)	0.49 (0.33–0.73)	
PACT team member (ref. is Yes)					
No	0.43 (0.34–0.53)	0.52 (0.41–0.64)	0.39 (0.31–0.5)	0.17 (0.13–0.21)	0.28 (0.22–0.36)
Not in teamlet	0.73 (0.58–0.9)		0.7 (0.57–0.88)	0.33 (0.26–0.42)	0.52 (0.41–0.64)
Not sure	0.32 (0.22–0.48)	0.39 (0.26–0.58)	0.28 (0.19–0.42)	0.22 (0.14–0.34)	0.31 (0.2–0.46)
Time in primary care (ref. is >80%)					
<20%	0.64 (0.49–0.84)	0.73 (0.57–0.94)	0.52 (0.39–0.68)		0.72 (0.55–0.94)
20%–40%					
41%–60%					
61%–80%					
**Covariate**	**Information systems**	**Scheduling tools**	**QI methods**	**Disease registries**	**Online toolkit**
Supervisor (versus not)	1.84 (1.6–2.12)	1.52 (1.32–1.75)	1.57 (1.39–1.77)	1.63 (1.42–1.88)	1.46 (1.3–1.65)
Time worked in VHA (ref. is <0.5 years)					
0.5–1 years					
1–2 years					
2–5 years		1.58 (1.12–2.25)	1.51 (1.06–2.14)	1.54 (1.08–2.18)	1.45 (1.02–2.05)
5–10 years		1.63 (1.13–2.36)	1.58 (1.09–2.29)	1.48 (1.02–2.14)	1.45 (1.02–2.05)
10–15 years		1.62 (1.12–2.34)	1.6 (1.11–2.32)	1.46 (1.01–2.12)	
15–20 years		1.86 (1.23–2.8)	1.6 (1.08–2.36)		1.62 (1.09–2.39)
>20 years	1.65 (1.09–2.48)	1.77 (1.2–2.61)	1.55 (1.05–2.29)	1.52 (1.03–2.25)	1.46 (1.01–2.12)
Role in primary care (ref. is provider)					
Administrative		1.63 (1.31–2.03)	1.38 (1.11–1.72)	0.45 (0.36–0.56)	
Dietician	0.39 (0.23–0.66)	0.47 (0.28–0.8)		0.38 (0.23–0.65)	
LPN/LVN/CNA	1.72 (1.38–2.14)	1.77 (1.45–2.16)	1.77 (1.45–2.16)		1.57 (1.31–1.88)
Mental health professional			0.5 (0.3–0.83)	0.28 (0.17–0.44)	0.49 (0.3–0.79)
Nurse care manager	1.45 (1.16–1.8)	1.23 (1.01–1.51)	1.45 (1.21–1.73)	1.3 (1.06–1.58)	1.51 (1.26–1.8)
Nurse case manager	1.45 (1.02–2.05)	1.46 (1.05–2.03)	1.4 (1.05–1.88)		1.4 (1.05–1.88)
Other		0.63 (0.39–1)			
Other RN	1.45 (1.08–1.93)		1.73 (1.35–2.23)		1.86 (1.42–2.44)
Pharmacist	0.63 (0.47–0.84)	0.53 (0.39–0.7)	0.74 (0.55–0.99)		0.71 (0.53–0.95)
Social worker	0.51 (0.35–0.74)	0.51 (0.35–0.74)		0.23 (0.16–0.35)	
Technician		2.12 (1.49–3)	1.82 (1.36–2.44)	0.69 (0.51–0.94)	1.34 (1–1.79)
PACT team member (ref. is Yes)					
No	0.4 (0.32–0.5)	0.4 (0.32–0.5)	0.78 (0.63–0.97)	0.61 (0.49–0.76)	0.72 (0.58–0.9)
Not in teamlet	0.72 (0.58–0.9)	0.7 (0.56–0.87)			
Not sure	0.35 (0.23–0.53)	0.44 (0.3–0.65)	0.59 (0.39–0.91)	0.47 (0.31–0.7)	0.43 (0.27–0.67)
Time in primary care (ref. is >80%)					
<20%				0.68 (0.52–0.9)	
20%–40%					
41%–60%					
61%–80%					

^1^Models are adjusted for age, sex, race, and facility complexity. See Additional file 1 for complete tables.

Relative to primary care providers, RN *care* managers (RNs working as a core member of a PACT team) were significantly more likely to use each of the ten resources (OR range, 1.23 to 2.14). RN *case* managers (RNs who provide condition-specific case management, such as diabetes, across teams) were more likely than providers to use four of the ten resources. LPNs, LVNs, and CNAs (OR range, 1.31 to 2.12) and other RNs (OR range, 1.34 to 1.93) also tended to be more likely to report resource use relative to primary care providers. Dieticians, mental health professionals, pharmacists, and social workers were less likely to report resource use than primary care providers.

For five of the ten resources, respondents who worked in primary care less than 20% of the time were significantly less likely (OR range, 0.52 to 0.73) to report use compared to those who worked in primary care more than 80% of the time. Age, race, and facility complexity were not consistently predictive of resource use/participation. See Additional file 1 for the complete listing of odds ratios.

### Significant predictors of utility among resource users

For eight of the ten resources, supervisors were less likely to rate the resource as being somewhat or very helpful (OR range, 0.72 to 0.84) than non-supervisors, after accounting for other covariates (Table 5). PACT team membership was not consistently associated with utility ratings. Longer tenure in the VHA tended to be positively associated with rating resources as helpful (OR range, 1.86 for “Scheduling tools” to 6.55 for “Online toolkit”).

**Table 5:** **Significant odds ratios (95% confidence intervals) for predictors of resource utility among resource users** ^**1**^

**Covariate**	**Local PACT education**	**PACT collaborative**	**Measures**	**Teamlet huddles**	**Teamlet meetings**
Supervisor (versus not)	0.76 (0.64–0.91)	0.72 (0.6–0.86)	0.79 (0.67–0.92)	0.74 (0.58–0.94)	0.72 (0.57–0.91)
Time worked in VHA (ref. is <0.5 years)					
0.5–1 years					
1–2 years	2.64 (1.19–5.87)	2.44 (1.12–5.31)	2.66 (1.28–5.53)		
2–5 years	4.18 (1.92–9.12)	3.78 (1.8–7.92)	3.39 (1.7–6.75)		2.69 (1.12–6.49)
5–10 years	4.95 (2.27–10.8)	4.14 (1.97–8.67)	4.06 (2.03–8.08)		2.69 (1.12–6.49)
10–15 years	4.48 (2.05–9.78)	4.22 (1.97–9.03)	2.97 (1.46–6.05)	2.44 (1.05–5.64)	3.1 (1.26–7.61)
15–20 years	4.85 (2.18–10.8)	4.26 (1.95–9.3)	3.56 (1.72–7.39)		2.94 (1.15–7.54)
>20 years	3.71 (1.67–8.25)	2.94 (1.38–6.3)	2.61 (1.26–5.42)		
Role in primary care (ref. is provider)					
Administrative	0.44 (0.32–0.59)	0.39 (0.29–0.54)	0.53 (0.39–0.7)	0.62 (0.42–0.91)	0.55 (0.37–0.81)
Dietician	0.05 (0.01–0.33)		0.14 (0.04–0.47)		
LPN/LVN/CNA	0.51 (0.39–0.65)	0.58 (0.45–0.75)	0.58 (0.45–0.73)	0.64 (0.46–0.9)	0.53 (0.38–0.74)
Mental health professional	0.39 (0.2–0.79)	0.44 (0.21–0.92)			
Nurse care manager	0.56 (0.44–0.71)	0.69 (0.54–0.88)	0.72 (0.58–0.9)	0.55 (0.39–0.76)	0.55 (0.4–0.77)
Nurse case manager	0.55 (0.37–0.84)		0.63 (0.43–0.93)		
Other	0.320.13–0.77)	0.34 (0.15–0.76)	0.3 (0.13–0.68)		
Other RN	0.39 (0.26–0.59)	0.57 (0.38–0.86)	0.54 (0.38–0.77)		0.45 (0.26–0.78)
Pharmacist	0.37 (0.24–0.57)	0.35 (0.22–0.54)	0.49 (0.32–0.73)		
Social worker	0.28 (0.15–0.53)	0.32 (0.17–0.62)	0.5 (0.28–0.9)	0.19 (0.06–0.64)	0.15 (0.04–0.51)
Technician	0.47 (0.29–0.75)	0.53 (0.34–0.83)	0.43 (0.27–0.69)		
PACT team member (ref. is Yes)					
No					
Not in teamlet	0.66 (0.47–0.91)	0.7 (0.51–0.98)			
Not sure	2.03 (1.11–3.74)	2.64 (1.38–5.05)	3.35 (1.75–6.42)	2.8 (1.4–5.58)	2.27 (1.08–4.76)
Time in primary care (ref. is >80%)					
<20%	0.59 (0.37–0.95)		0.44 (0.27–0.72)		
20%–40%					
41%–60%	0.58 (0.36–0.92)				
61%–80%	0.61 (0.4–0.91)				
**Covariate**	**Information systems**	**Scheduling tools**	**QI methods**	**Disease registries**	**Online toolkit**
Supervisor (versus not)	0.84 (0.7–1)		0.72 (0.58–0.9)		0.76 (0.63–0.93)
Time worked in VHA (ref. is <0.5 years)					
0.5–1 years					
1–2 years	2.86 (1.28–6.36)				2.92 (1.05–8.08)
2–5 years	4.53 (2.12–9.68)	1.88 (1.04–3.39)	2.97 (1.31–6.75)	3.9 (1.52–9.97)	5.81 (2.18–15.5)
5–10 years	4.35 (1.99–9.49)	1.86 (1.03–3.35)	2.94 (1.3–6.69)	4.18 (1.63–10.7)	6.55 (2.46–17.5)
10–15 years	3.9 (1.79–8.5)	1.88 (1.02–3.46)	3.63 (1.6–8.25)	3.67 (1.4–9.58)	6.49 (2.39–17.6)
15–20 years	4.06 (1.82–9.03)		3.1 (1.31–7.32)	3.67 (1.38–9.78)	5.93 (2.18–16.1)
>20 years	2.51 (1.13–5.58)				4.48 (1.65–12.2)
Role in primary care (ref. is provider)					
Administrative	0.42 (0.31–0.58)	0.72 (0.54–0.96)	0.31 (0.21–0.44)	0.61 (0.42–0.89)	0.35 (0.24–0.51)
Dietician	0.19 (0.04–0.84)		0.11 (0.02–0.54)		0.05 (0.01–0.41)
LPN/LVN/CNA	0.54 (0.41–0.71)	0.73 (0.57–0.93)	0.45 (0.33–0.61)	0.48 (0.35–0.65)	0.47 (0.34–0.64)
Mental health professional					0.35 (0.13–0.92)
Nurse care manager	0.68 (0.53–0.86)		0.59 (0.44–0.79)	0.63 (0.48–0.82)	0.55 (0.42–0.73)
Nurse case manager	0.61 (0.41–0.92)		0.49 (0.3–0.8)		0.44 (0.27–0.73)
Other	0.26 (0.1–0.72)				0.31 (0.13–0.73)
Other RN	0.51 (0.34–0.76)		0.49 (0.32–0.75)	0.64 (0.41–1)	0.48 (0.31–0.74)
Pharmacist	0.39 (0.24–0.63)	0.59 (0.37–0.94)	0.41 (0.23–0.7)	0.3 (0.17–0.53)	0.22 (0.12–0.4)
Social worker	0.5 (0.25–1)	0.44 (0.22–0.9)	0.36 (0.16–0.78)		0.2 (0.08–0.47)
Technician	0.51 (0.31–0.84)	0.57 (0.37–0.88)	0.39 (0.23–0.64)		0.46 (0.28–0.77)
PACT team member (ref. is Yes)					
No					0.63 (0.4–0.98)
Not in teamlet					
Not sure	3.78 (1.97–7.24)	2.59 (1.4–4.76)	3.03 (1.31–7.03)	2.53 (1.25–5.16)	3.06 (1.3–7.24)
Time in primary care (ref. is >80%)					
<20%	0.59 (0.37–0.95)		0.52 (0.32–0.84)		
20%–40%	0.54 (0.3–1)				
41%–60%					
61%–80%			0.61 (0.38–0.97)		

^1^Models are adjusted for age, sex, race, and facility complexity. See Additional file 2 for complete tables.

Across all ten models, respondents working in administrative or clinical associate roles were less likely to find the resources helpful (OR range, 0.31 for “QI methods” to 0.73 for “Scheduling tools”) than providers. For eight of the ten resources, nurse care managers were less likely to rate the resources as helpful than providers. Overall, the time spent working in primary care was not consistently associated with utility ratings. Facility complexity was not significantly associated with utility ratings. See Additional file 2 for the complete listing of odds ratios.

## Discussion

Implementing a PCMH is a fundamental redesign of primary care, and we know of few efforts to systematically rate the use and utility of the implementation resources. As this model continues to spread, health systems can benefit from knowing how to package and deliver implementation-support resources to members of PCMH teams [24-26], something relatively little is known about [27].

Teamlet huddles were the most used and most helpful resource. These brief meetings allow PCMH team members to talk about and plan for the daily management of patient demands and clinic workflow, address specific patients’ needs and preferences, and improve the delivery of preventive services [28]. In at least one other study, huddles have been found to be associated with better teamwork and an improved practice environment [28]. The informal nature of these meetings and the fact that they are temporally connected to the day’s workflow may make this especially salient to busy primary care staff.

Quality-improvement methods to conduct small tests of change were the least used, although over 40% of the respondents reported using QI methods, and two-thirds of the users found them to be helpful. PCMH measurement tools were widely used but less often rated as helpful. These findings are striking in light of the centrality of Plan-Do-Study-Act (PDSA) and related quality measurement approaches in many primary care systems and PCMH models. It may be that greater training and/or experience is needed before PDSA and similar approaches are useful. Although not part of this analysis, some VHA regional networks did provide internal coaches. Internal coaches may have been especially helpful in assisting staff on the use of quality-improvement tools and methods and in helping staff see the applied value. We also did not assess the use of more comprehensive approaches to PACT implementation, such as practice facilitation, which has been found to be effective for guideline implementation in other settings [29]. In general, PCMH-implementation leaders may want to consider training and dissemination strategies that emphasize the linkage between measurement activities and PCMH effectiveness to boost uptake. Regular teamlet meetings to discuss improvement were also widely used and perceived as helpful, but patterns of use and utility were less clear than for other resources. Although the online toolkit of care delivery and organization tools was not as widely used or highly rated as the other resources, it represents a relatively inexpensive and “on-demand” resource to facilitate the sharing of best practices across disparate sites [30]. PCMH leaders should consider how to best disseminate online toolkits in the context of their own health system.

Individuals working in administrative roles were more likely than providers to have used or participated in activities intended to improve clinic access (e.g., through proactive scheduling) and quality-improvement methods to conduct small tests of change. However, they were less likely to rate those resources as being helpful than providers. If, in fact, administrative staff is the most appropriate audience for those particular resources, our data suggests there may be a need to revise their content, format, or delivery. On the other hand, if administrative staff is not the intended audience, it may be helpful to clarify for those working in primary care the purpose and intended audience of these resources. Although supervisors were more likely to have used or participated in the various PACT-related activities or resources than non-supervisors, they were less likely to rate them “helpful.” There are several possible reasons for that, including the fact that supervisors may not actually have been learning anything new or that the resources were not optimized to their job functions. Those working in supervisory roles may not have had the ability to “opt out” of participation in certain activities, making them more likely to be exposed regardless of how helpful they found the activities to be. In contrast, support staff may have self-selected those activities and resources they found most appealing. Supervisors may also be more comfortable than non-supervisors in giving a frank appraisal of resources or may simply have more time to devote to exploring new resources than front-line staff.

Although individuals working in nursing roles were consistently more likely to have used the resources or participated in the activities than providers, they were less likely to rate them as being “helpful.” The fact that providers who rated the resources and activities were consistently more likely to rate them as “useful” than other users suggests that there may be a need for additional customization of the intervention-boosting resources if we intend for them to appeal to a more diverse group of individuals.

Depending on roles and responsibilities, individuals may engage differently during the implementation of complex interventions like PCMH. In their analysis of survey data from primary care staff who participated in a virtual learning collaborative, Butler et al. found that those who benefited most from the collaborative were those with prior PCMH training and those who fully engaged in collaborative activities though non-providers and newcomers to the PCMH model found the collaboratives to be less helpful [31]. Similarly, in their qualitative analysis of interviews on PCMH implementation among staff delivering care to older adults, Hoff et al. found that a single, across-the-board implementation approach may not be effective [32]. Having a cadre of implementation resources may be valuable, and knowing what resources to make available when selectively targeting certain groups of individuals may be a more efficient and effective means of ensuring uptake. Our data provides a framework for guiding those decisions.

Facility complexity was of limited predictive utility, which may indicate that more person and workgroup targeted implementation-boosting efforts is warranted.

There are limitations to our cross-sectional study. First, there are important limitations to the generalizability of our findings. The VHA’s highly integrated structure may have contributed to the ease with which a PCMH model could be implemented. Similarly, this may have made it easier to disseminate the resources. Outside the VHA, economic incentives, competition, and other external forces may serve to facilitate PCMH uptake [4], which could not be assessed in this study. Second, there is potential measurement bias related to the survey items which were developed for the PCMH evaluation. A number of the resources identified in the survey had similar sounding names. Respondents may not have been able to accurately differentiate between them resulting in some inaccurate ratings or measurement errors. For example, “Teamlet huddles” versus “Regular (non-huddles) teamlet meetings.” Third, there is potential selection bias. This survey was distributed through clinical leadership and was anonymous and voluntary; therefore, we do not have a defined sample from which to derive a response rate or detect response bias. However, we were able to compare our respondent demographics to primary care respondents to a separate survey fielded annually to all VHA employees, which in 2012 achieved a 62% response rate, and our respondents’ demographics were very similar; our sample had a higher proportion of supervisors and fewer African-Americans, among the four occupations comprising PACTs [21]. Our sample was also representative of primary care clinics, with 69% of VHA primary care sites nationally being represented in our findings. Future research correlating timing of the availability of activities and resources with the larger implementation efforts and outcomes would add value to these types of evaluations.

Future assessments of the use and uptake of implementation-boosting resources would be enhanced by examining trends in use over time. For example, some activities and resources may be most valuable when implementation is beginning while others are important to the long-term sustainability of whatever is being implemented. This information could help inform the allocation of financial and personnel resources needed at the onset to support implementation versus resources needed several weeks or months down the line. Additionally, knowing where the system is most likely to face challenges in adopting new care delivery models or quality programs will inform the customization, timing, and marketing of these types of resources.

## Conclusion

How to best implement and spread complex quality improvement and system redesign initiatives like the patient-centered medical home is a challenge. These data shed light on a little-studied but crucial question: What resources do staff find most helpful in implementing PCMH? Teamlet huddles were the most frequently used and liked resources while QI methods to conduct small tests of change, though often promoted in PCMH models, were least frequently used although found helpful by a majority using them. Our data support targeted outreach to those less invested in the host system adopting the innovation, such as those with shorter tenure or in supporting roles. Such outreach will likely be even more important outside of integrated systems where institutional loyalties may be more fragmented. Our analysis of the availability of implementation-boosting resources during the VHA’s rollout is a useful model for policymakers and systems redesign staff charged with setting priorities for the design and dissemination of complex initiatives.

## Additional files

Additional file 1:
**Predictors of resource use.** Complete table of all predictors (significant and not significant) of resource use. Additional file 2:
**Predictors of resource utility among resource users.** Complete table of all predictors (significant and not significant) of resource utility among resource users.
