# Supplementary material for: The most used and most helpful facilitators for patient-centered medical home implementation
Source: Implement Sci. 2015 Apr 19;10:52. doi: 10.1186/s13012-015-0246-9 (PMC4414441; doi:10.1186/s13012-015-0246-9)
Supplement: Additional file 1: — Predictors of resource use. Complete table of all predictors (significant and not significant) of resource use. [file 13012_2015_246_MOESM1_ESM.docx]

Predictors of resource use, odds ratio (95% confidence interval)

| Covariate | Local PACT education | PACT collaborative | Measures | Teamlet huddles | | Teamlet meetings |
| --- | --- | --- | --- | --- | --- | --- |
| Supervisor (versus not) | 1.68 (1.43–1.97) | 1.55 (1.35–1.79) | 1.86 (1.58–2.18) | 1.63 (1.36–1.95) | | 1.63 (1.39–1.92) |
| Time worked in VHA (ref. is <0.5 years) | | | | | | |
| 0.5–1 years | 1.2 (0.78–1.84) | 0.95 (0.63–1.43) | 1.39 (0.89–2.18) | 0.79 (0.46–1.34) | | 0.97 (0.61–1.55) |
| 1–2 years | 1.86 (1.26–2.75) | 1.31 (0.9–1.9) | 1.95 (1.3–2.94) | 1.25 (0.76–2.03) | | 1.04 (0.68–1.6) |
| 2–5 years | 2.08 (1.43–3.0) | 1.58 (1.12–2.25) | 2.14 (1.48–3.1) | 1.32 (0.84–2.08) | | 1.02 (0.69–1.51) |
| 5–10 years | 1.77 (1.22–2.56) | 1.57 (1.11–2.23) | 1.93 (1.34–2.8) | 1.07 (0.68–1.68) | | 1.03 (0.7–1.52) |
| 10–15 years | 2.08 (1.4–3.06) | 1.67 (1.15–2.41) | 1.75 (1.19–2.59) | 1.2 (0.73–1.95) | | 1.07 (0.71–1.62) |
| 15–20 years | 2.03 (1.35–3.06) | 1.68 (1.14–2.48) | 1.92 (1.25–2.94) | 1.35 (0.81–2.25) | | 1.36 (0.87–2.14) |
| >20 years | 2.64 (1.75–3.97) | 1.68 (1.16–2.44) | 2.39 (1.58–3.6) | 1.22 (0.75–1.99) | | 1.16 (0.76–1.79) |
| Respondent ethnicity (ref. is White) | | | | | | |
| Black/African- American | 0.96 (0.76–1.22) | 1.14 (0.91–1.42) | 1.05 (0.83–1.34) | 1.01 (0.77–1.32) | | 1.17 (0.92–1.49) |
| Asian/Pacific Islander | 1.23 (0.92–1.65) | 1.32 (1.03–1.7) | 1.17 (0.88–1.57) | 1.12 (0.8–1.55) | | 1.65 (1.23–2.2) |
| Hispanic | 0.97 (0.74–1.27) | 1.09 (0.85–1.4) | 0.91 (0.7–1.2) | 1.05 (0.76–1.46) | | 1.07 (0.82–1.4) |
| Other | 0.87 (0.68–1.12) | 0.92 (0.73–1.17) | 0.91 (0.7–1.2) | 0.86 (0.63–1.17) | | 1.12 (0.85–1.46) |
| Respondent age (ref is ≥60) | | | | | | |
| <20–29 | 1.01 (0.7–1.46) | 0.9 (0.65–1.26) | 1.07 (0.74–1.55) | 1.04 (0.68–1.6) | | 1.16 (0.8–1.68) |
| 30–39 | 0.87 (0.68–1.12) | 0.86 (0.38–1.09) | 1.03 (0.8–1.32) | 0.87 (0.64–1.19) | | 0.9 (0.7–1.16) |
| 40–49 | 0.97 (0.76–1.23) | 0.91 (0.75–1.12) | 1.04 (0.82–1.32) | 1.04 (0.79–1.36) | | 1.08 (0.85–1.38) |
| 50–59 | 1.03 (0.83–1.28) | 1.04 (0.85–1.27) | 1.14 (0.91–1.42) | 1.01 (0.77–1.32) | | 1.05 (0.84–1.31) |
| Female sex (versus male) | 0.82 (0.7–0.96) | 0.73 (0.64–0.84) | 0.64 (0.55–0.76) | 0.71 (0.58–0.87) | | 0.73 (0.63–0.86) |
| Role in primary care (ref. is provider) | | | | | | |
| Administrative | 0.77 (0.61–0.98) | 1.2 (0.96–1.49) | 0.85 (0.67–1.08) | 0.7 (0.54–0.92) | | 1.28 (1.01–1.63) |
| Dietician | 0.54 (0.32–0.92) | 0.7 (0.42–1.17) | 0.43 (0.25–0.73) | 0.15 (0.09–0.27) | | 0.52 (0.3–0.9) |
| LPN/LVN/CNA | 1.31 (1.05–1.63) | 1.8 (1.48–2.2) | 1.48 (1.16–1.88) | 1.21 (0.92–1.58) | | 2.12 (1.67–2.69) |
| Mental health professional | 1.28 (0.79–2.1) | 0.93 (0.61–1.43) | 0.39 (0.25–0.62) | 0.46 (0.28–0.76) | | 0.89 (0.55–1.42) |
| Nurse care manager | 1.3 (1.04–1.62) | 1.42 (1.16–1.73) | 1.52 (1.2–1.93) | 2.14 (1.57–2.92) | | 1.86 (1.49–2.32) |
| Nurse case manager | 1.4 (0.95–2.08) | 1.06 (0.78–1.45) | 1.19 (0.82–1.72) | 1.02 (0.65–1.6) | | 1.32 (0.93–1.88) |
| Other | 0.94 (0.58–1.54) | 1.43 (0.88–2.34) | 0.7 (0.43–1.15) | 0.39 (0.23–0.65) | | 0.92 (0.57–1.51) |
| Other RN | 1.26 (0.92–1.72) | 1.34 (1.02–1.75) | 1.34 (0.96–1.86) | 1.25 (0.86–1.8) | | 1.93 (1.42–2.64) |
| Pharmacist | 0.79 (0.58–1.07) | 1.07 (0.8–1.43) | 0.57 (0.42–0.78) | 0.19 (0.14–0.27) | | 0.78 (0.57–1.06) |
| Social worker | 0.91 (0.62–1.35) | 1.07( 0.74–1.55) | 0.44 (0.31–0.64) | 0.38 (0.25–0.58) | | 0.85 (0.59–1.23) |
| Technician | 0.88 (0.63–1.22) | 1.22 (0.9–1.67) | 0.68 (0.49–0.95) | 0.49 (0.33–0.73) | | 1.26 (0.89–1.79) |
| PACT team member (ref. is Yes) | | | | | | |
| No | 0.43 (0.34–0.53) | 0.52 (0.41–0.64) | 0.39 (0.31–0.5) | 0.17 (0.13–0.21) | | 0.28 (0.22–0.36) |
| Not in teamlet | 0.73 (0.58–0.9) | 0.9 (0.74–1.11) | 0.7 (0.57–0.88) | 0.33 (0.26–0.42) | | 0.52 (0.41–0.64) |
| Not sure | 0.32 (0.22–0.48) | 0.39 (0.26–0.58) | 0.28 (0.19–0.42) | 0.22 (0.14–0.34) | | 0.31 (0.2–0.46) |
| Time in primary care (ref. is >80%) | | | | | | |
| <20% | 0.64 (0.49–0.84) | 0.73 (0.57–0.94) | 0.52 (0.39–0.68) | 0.82 (0.61–1.09) | | 0.72 (0.55–0.94) |
| 20%–40% | 1.28 (0.82–2.01) | 1.11 (0.76–1.6) | 0.95 (0.63–1.43) | 0.94 (0.6–1.48) | | 0.93 (0.63–1.38) |
| 41%–60% | 1.43 (0.97–2.12) | 1.27 (0.91–1.77) | 1.07 (0.76–1.52) | 0.91 (0.62–1.35) | | 1.09 (0.77–1.55) |
| 61%–80% | 1.05 (0.77–1.43) | 1.06 (0.81–1.39) | 1 (0.73–1.36) | 0.81 (0.57–1.15) | | 0.76 (0.57–1.02) |
| Facility complexity (ref. is “1” or “most complex”) | | | | |  |  |
| 2 | 0.74 (0.58–0.95) | 0.87 (0.68–1.11) | 0.86 (0.64–1.15) | 0.73 (0.53–1.02) | | 0.65 (0.5–0.85) |
| 3 | 0.93 (0.71–1.22) | 0.82 (0.64–1.04) | 1.08 (0.79–1.48) | 1.19 (0.82–1.72) | | 0.89 (0.66–1.19) |

Predictors of resource use, odds ratio (95% confidence interval), continued

| Covariate | Information systems | Scheduling tools | QI methods | Disease registries | | Online toolkit |
| --- | --- | --- | --- | --- | --- | --- |
| Supervisor (versus not) | 1.84 (1.6–2.12) | 1.52 (1.32–1.75) | 1.57 (1.39–1.77) | 1.63 (1.42–1.88) | | 1.46 (1.3–1.65) |
| Time worked in VHA (ref. is <0.5 years) | | | | | | |
| 0.5–1 years | 0.9 (0.58–1.38) | 0.84 (0.56–1.27) | 0.96 (0.63–1.48) | 1.08 (0.7–1.67) | | 1.06 (0.7–1.6) |
| 1–2 years | 1.25 (0.84–1.84) | 1.46 (0.99–2.16) | 1.3 (0.88–1.92) | 1.16 (0.79–1.72) | | 1.23 (0.85–1.79) |
| 2–5 years | 1.4 (0.97–2.03) | 1.58 (1.12–2.25) | 1.51 (1.06–2.14) | 1.54 (1.08–2.18) | | 1.45 (1.02–2.05) |
| 5–10 years | 1.3 (0.9–1.88) | 1.63 (1.13–2.36) | 1.58 (1.09–2.29) | 1.48 (1.02–2.14) | | 1.45 (1.02–2.05) |
| 10–15 years | 1.42 (0.96–2.1) | 1.62 (1.12–2.34) | 1.6 (1.11–2.32) | 1.46 (1.01–2.12) | | 1.42 (0.98–2.05) |
| 15–20 years | 1.46 (0.97–2.2) | 1.86 (1.23–2.8) | 1.6 (1.08–2.36) | 1.39 (0.94–2.05) | | 1.62 (1.09–2.39) |
| >20 years | 1.65 (1.09–2.48) | 1.77 (1.2–2.61) | 1.55 (1.05–2.29) | 1.52 (1.03–2.25) | | 1.46 (1.01–2.12) |
| Respondent ethnicity (ref. is White) | | | | | | |
| Black/African-American | 1.05 (0.83–1.34) | 1.03 (0.83–1.28) | 1.19 (0.97–1.45) | 1.22 (0.98–1.52) | | 1.03 (0.84–1.26) |
| Asian/Pacific Islander | 1.34 (1.02–1.75) | 1.51 (1.17–1.93) | 1.88 (1.51–2.34) | 1.52 (1.19–1.95) | | 1.35 (1.08–1.68) |
| Hispanic | 0.98 (0.76–1.26) | 1.07 (0.84–1.38) | 1.31 (1.03–1.67) | 1.27 (0.99–1.63) | | 1.19 (0.93–1.51) |
| Other | 1.04 (0.81–1.34) | 1.01 (0.79–1.28) | 1.21 (0.97–1.51) | 1.21 (0.95–1.54) | | 1.17 (0.94–1.46) |
| Respondent age (ref is ≥60) | | | | | | |
| <20–29 | 1.08 (0.76–1.54) | 1.39 (1–1.93) | 1.11 (0.79–1.54) | 1.05 (0.76–1.46) | | 0.79 (0.57–1.09) |
| 30–39 | 0.8 (0.63–1.02) | 1.03 (0.81–1.31) | 1.06 (0.85–1.32) | 1 (0.79–1.27) | | 0.9 (0.72–1.12) |
| 40–49 | 0.85 (0.68–1.06) | 1.19 (0.97–1.45) | 1.08 (0.89–1.32) | 1.01 (0.83–1.23) | | 1.01 (0.83–1.23) |
| 50–59 | 1.05 (0.84–1.31) | 1.21 (0.99–1.48) | 1.08 (0.9–1.3) | 0.98 (0.8–1.2) | | 1.03 (0.86–1.23) |
| Female sex (versus male) | 0.66 (0.56–0.77) | 0.79 (0.69–0.91) | 0.58 (0.51–0.67) | 0.7 (0.61–0.8) | | 0.65 (0.57–0.75) |
| Role in primary care (ref. is provider) | | | | | | |
| Administrative | 1.09 (0.88–1.36) | 1.63 (1.31–2.03) | 1.38 (1.11–1.72) | 0.45 (0.36–0.56) | | 1 (0.82–1.22) |
| Dietician | 0.39 (0.23–0.66) | 0.47 (0.28–0.8) | 0.7 (0.41–1.22) | 0.38 (0.23–0.65) | | 0.7 (0.41–1.19) |
| LPN/LVN/CNA | 1.72 (1.38–2.14) | 1.77 (1.45–2.16) | 1.77 (1.45–2.16) | 1.2 (0.98–1.46) | | 1.57 (1.31–1.88) |
| Mental health professional | 0.66 (0.42–1.03) | 0.79 (0.52–1.22) | 0.5 (0.3–0.83) | 0.28 (0.17–0.44) | | 0.49 (0.3–0.79) |
| Nurse care manager | 1.45 (1.16–1.8) | 1.23 (1.01–1.51) | 1.45 (1.21–1.73) | 1.3 (1.06–1.58) | | 1.51 (1.26–1.8) |
| Nurse case manager | 1.45 (1.02–2.05) | 1.46 (1.05–2.03) | 1.4 (1.05–1.88) | 1.23 (0.9–1.68) | | 1.4 (1.05–1.88) |
| Other | 0.89 (0.54–1.45) | 0.63 (0.39–1) | 0.95 (0.59–1.52) | 0.84 (0.53–1.35) | | 1.21 (0.76–1.93) |
| Other RN | 1.45 (1.08–1.93) | 1.12 (0.85–1.46) | 1.73 (1.35–2.23) | 1.04 (0.79–1.36) | | 1.86 (1.42–2.44) |
| Pharmacist | 0.63 (0.47–0.84) | 0.53 (0.39–0.7) | 0.74 (0.55–0.99) | 0.89 (0.66–1.19) | | 0.71 (0.53–0.95) |
| Social worker | 0.51 (0.35–0.74) | 0.51 (0.35–0.74) | 0.78 (0.53–1.15) | 0.23 (0.16–0.35) | | 0.81 (0.56–1.17) |
| Technician | 1.08 (0.78–1.51) | 2.12 (1.49–3) | 1.82 (1.36–2.44) | 0.69 (0.51–0.94) | | 1.34 (1–1.79) |
| PACT team member (ref. is Yes) | | | | | | |
| No | 0.4 (0.32–0.5) | 0.4 (0.32–0.5) | 0.78 (0.63–0.97) | 0.61 (0.49–0.76) | | 0.72 (0.58–0.9) |
| Not in Teamlet | 0.72 (0.58–0.9) | 0.7 (0.56–0.87) | 1.21 (0.99–1.48) | 0.95 (0.76–1.19) | | 1.16 (0.95–1.42) |
| Not sure | 0.35 (0.23–0.53) | 0.44 (0.3–0.65) | 0.59 (0.39–0.91) | 0.47 (0.31–0.7) | | 0.43 (0.27–0.67) |
| Time in primary care (ref. is >80%) | | | | | | |
| <20% | 0.79 (0.6–1.03) | 0.79 (0.61–1.04) | 1.05 (0.82–1.35) | 0.68 (0.52–0.9) | | 0.81 (0.63–1.04) |
| 20%–40% | 0.84 (0.57–1.23) | 1.04 (0.72–1.51) | 1.4 (0.99–1.99) | 1.39 (0.94–2.05) | | 1.28 (0.9–1.82) |
| 41%–60% | 0.91 (0.66–1.27) | 1.03 (0.76–1.4) | 1.3 (0.95–1.77) | 0.96 (0.7–1.31) | | 1.13 (0.83–1.54) |
| 61%–80% | 0.91 (0.68–1.22) | 1.21 (0.9–1.62) | 1.13 (0.86–1.48) | 1.05 (0.79–1.4) | | 1.09 (0.84–1.43) |
| Facility complexity (ref. is “1” or “most complex”) | | | | |  |  |
| 2 | 0.78 (0.61–1) | 1 (0.79–1.27) | 0.91 (0.75–1.12) | 0.9 (0.7–1.15) | | 0.89 (0.73–1.08) |
| 3 | 1.04 (0.79–1.36) | 1.04 (0.81–1.34) | 1.2 (0.96–1.49) | 1.23 (0.94–1.62) | | 1.12 (0.9–1.39) |
