# Supplementary material for: The most used and most helpful facilitators for patient-centered medical home implementation
Source: Implement Sci. 2015 Apr 19;10:52. doi: 10.1186/s13012-015-0246-9 (PMC4414441; doi:10.1186/s13012-015-0246-9)
Supplement: Additional file 2: — Predictors of resource utility among resource users. Complete table of all predictors (significant and not significant) of resource utility among resource users. [file 13012_2015_246_MOESM2_ESM.docx]

Predictors of resource utility among resource users, odds ratio (95% confidence interval)

| Covariate | Local PACT education | PACT collaborative | Measures | Teamlet huddles | | Teamlet meetings |
| --- | --- | --- | --- | --- | --- | --- |
| Supervisor (versus not) | 0.76 (0.64–0.91) | 0.72 (0.6–0.86) | 0.79 (0.67–0.92) | 0.74 (0.58–0.94) | | 0.72 (0.57–0.91) |
| Time worked in VHA (ref. is <0.5 years) | | | | | | |
| 0.5–1 years | 1.86 (0.77–4.48) | 1.39 (0.58–3.35) | 1.73 (0.79–3.78) | 1.12 (0.41–3.03) | | 1.67 (0.6–4.62) |
| 1–2 years | 2.64 (1.19–5.87) | 2.44 (1.12–5.31) | 2.66 (1.28–5.53) | 1.19 (0.49–2.86) | | 1.34 (0.51–3.49) |
| 2–5 years | 4.18 (1.92–9.12) | 3.78 (1.8–7.92) | 3.39 (1.7–6.75) | 2.08 (0.91–4.71) | | 2.69 (1.12–6.49) |
| 5–10 years | 4.95 (2.27–10.8) | 4.14 (1.97–8.67) | 4.06 (2.03–8.08) | 1.9 (0.82–4.39) | | 2.69 (1.12–6.49) |
| 10–15 years | 4.48 (2.05–9.78) | 4.22 (1.97–9.03) | 2.97 (1.46–6.05) | 2.44 (1.05–5.64) | | 3.1 (1.26–7.61) |
| 15–20 years | 4.85 (2.18–10.8) | 4.26 (1.95–9.3) | 3.56 (1.72–7.39) | 1.95 (0.81–4.71) | | 2.94 (1.15–7.54) |
| >20 years | 3.71 (1.67–8.25) | 2.94 (1.38–6.3) | 2.61 (1.26–5.42) | 1.95 (0.83–4.62) | | 2.14 (0.84–5.47) |
| Respondent ethnicity (ref. is White) | | | | | | |
| Black/African-American | 0.57 (0.41–0.79) | 0.58 (0.42–0.79) | 0.49 (0.36–0.67) | 0.43 (0.27–0.68) | | 0.53 (0.35–0.82) |
| Asian/Pacific Islander | 0.5 (0.36–0.7) | 0.51 (0.37–0.71) | 0.61 (0.46–0.82) | 0.64 (0.42–0.99) | | 0.57 (0.38–0.86) |
| Hispanic | 0.84 (0.6–1.16) | 0.84 (0.6–1.16) | 0.73 (0.53–0.99) | 0.63 (0.39–1.01) | | 0.66 (0.41–1.05) |
| Other | 0.84 (0.61–1.14) | 0.84 (0.61–1.14) | 0.97 (0.73–1.3) | 0.99 (0.67–1.46) | | 0.98 (0.66–1.45) |
| Respondent age (ref is ≥60) | | | | | | |
| <20–29 | 2.14 (1.34–3.42) | 1.38 (0.84–2.25) | 1.26 (0.8–1.97) | 1.88 (0.96–3.67) | | 1.46 (0.75–2.86) |
| 30–39 | 2.1 (1.54–2.86) | 1.36 (1–1.86) | 1.4 (1.05–1.88) | 1.84 (1.2–2.83) | | 1.67 (1.08–2.56) |
| 40–49 | 1.65 (1.26–2.16) | 1.21 (0.92–1.58) | 1.17 (0.91–1.51) | 1.79 (1.23–2.59) | | 1.84 (1.27–2.66) |
| 50–59 | 1.26 (0.98–1.62) | 1.02 (0.79–1.31) | 1.17 (0.92–1.49) | 1.54 (1.06–2.23) | | 1.16 (0.82–1.65) |
| Female sex (versus male) | 0.68 (0.57–0.82) | 0.61 (0.51–0.73) | 0.71 (0.59–0.85) | 0.68 (0.53–0.86) | | 0.72 (0.57–0.91) |
| Role in primary care (ref. is provider) | | | | | | |
| Administrative | 0.44 (0.32–0.59) | 0.39 (0.29–0.54) | 0.53 (0.39–0.7) | 0.62 (0.42–0.91) | | 0.55 (0.37–0.81) |
| Dietician | 0.05 (0.01–0.33) | 0 (0–Inf) | 0.14 (0.04–0.47) | 0.52 (0.11–2.39) | | 0 (0–Inf) |
| LPN/LVN/CNA | 0.51 (0.39–0.65) | 0.58 (0.45–0.75) | 0.58 (0.45–0.73) | 0.64 (0.46–0.9) | | 0.53 (0.38–0.74) |
| Mental health professional | 0.39 (0.2–0.79) | 0.44 (0.21–0.92) | 0.71 (0.36–1.42) | 0.31 (0.09–1.05) | | 0.35 (0.12–1.04) |
| Nurse care manager | 0.56 (0.44–0.71) | 0.69 (0.54–0.88) | 0.72 (0.58–0.9) | 0.55 (0.39–0.76) | | 0.55 (0.4–0.77) |
| Nurse case manager | 0.55 (0.37–0.84) | 0.83 (0.55–1.25) | 0.63 (0.43–0.93) | 0.64 (0.36–1.14) | | 0.62 (0.36–1.07) |
| Other | 0.32 (0.13–0.77) | 0.34 (0.15–0.76) | 0.3 (0.13–0.68) | 0.39 (0.11–1.4) | | 0.33 (0.09–1.2) |
| Other RN | 0.39 (0.26–0.59) | 0.57 (0.38–0.86) | 0.54 (0.38–0.77) | 0.65 (0.39–1.08) | | 0.45 (0.26–0.78) |
| Pharmacist | 0.37 (0.24–0.57) | 0.35 (0.22–0.54) | 0.49 (0.32–0.73) | 0.86 (0.48–1.55) | | 0.68 (0.39–1.17) |
| Social Worker | 0.28 (0.15–0.53) | 0.32 (0.17–0.62) | 0.5 (0.28–0.9) | 0.19 (0.06–0.64) | | 0.15 (0.04–0.51) |
| Technician | 0.47 (0.29–0.75) | 0.53 (0.34–0.83) | 0.43 (0.27–0.69) | 0.74 (0.41–1.34) | | 0.78 (0.45–1.35) |
| PACT team member (ref. is Yes) | | | | | | |
| No | 1.08 (0.76–1.54) | 0.96 (0.66–1.39) | 0.9 (0.63–1.27) | 0.96 (0.57–1.63) | | 0.84 (0.49–1.46) |
| Not in Teamlet | 0.66 (0.47–0.91) | 0.7 (0.51–0.98) | 0.88 (0.66–1.17) | 0.84 (0.52–1.34) | | 0.66 (0.41–1.06) |
| Not sure | 2.03 (1.11–3.74) | 2.64 (1.38–5.05) | 3.35 (1.75–6.42) | 2.8 (1.4–5.58) | | 2.27 (1.08–4.76) |
| Time in primary care (ref. is >80%) | | | | | | |
| <20% | 0.59 (0.37–0.95) | 0.76 (0.48–1.19) | 0.44 (0.27–0.72) | 1.49 (0.91–2.44) | | 1.12 (0.64–1.93) |
| 20%–40% | 1.22 (0.76–1.95) | 1.15 (0.7–1.88) | 0.76 (0.46–1.23) | 0.86 (0.42–1.75) | | 1.3 (0.69–2.44) |
| 41%–60% | 0.58 (0.36–0.92) | 0.72 (0.45–1.15) | 0.96 (0.64–1.45) | 0.88 (0.48–1.62) | | 0.7 (0.38–1.32) |
| 61%–80% | 0.61 (0.4–0.91) | 0.92 (0.63–1.36) | 1.14 (0.8–1.62) | 0.73 (0.41–1.28) | | 0.82 (0.47–1.42) |
| Facility complexity (ref. is “1” or “most complex”) | | | | |  |  |
| 2 | 1.25 (0.9–1.73) | 1.08 (0.83–1.42) | 1.2 (0.91–1.57) | 1.16 (0.8–1.68) | | 1.17 (0.81–1.7) |
| 3 | 1.09 (0.77–1.55) | 1.02 (0.76–1.36) | 1.03 (0.77–1.38) | 0.79 (0.52–1.19) | | 1.25 (0.84–1.84) |

Predictors of resource utility among resource users, odds ratio (95% confidence interval), continued

| Covariate | Information systems | Scheduling tools | QI methods | Disease registries | | Online toolkit |
| --- | --- | --- | --- | --- | --- | --- |
| Supervisor (versus not) | 0.84 (0.7–1) | 0.87 (0.73–1.04) | 0.72 (0.58–0.9) | 0.9 (0.74–1.11) | | 0.76 (0.63–0.93) |
| Time worked in VHA (ref. is <0.5 years) | | | | | | |
| 0.5–1 years | 1.67 (0.68–4.1) | 0.92 (0.44–1.92) | 1.34 (0.51–3.49) | 1.86 (0.63–5.47) | | 1.72 (0.56–5.26) |
| 1–2 years | 2.86 (1.28–6.36) | 1.09 (0.58–2.05) | 2.16 (0.93–5) | 2.12 (0.79–5.64) | | 2.92 (1.05–8.08) |
| 2–5 years | 4.53 (2.12–9.68) | 1.88 (1.04–3.39) | 2.97 (1.31–6.75) | 3.9 (1.52–9.97) | | 5.81 (2.18–15.5) |
| 5–10 years | 4.35 (1.99–9.49) | 1.86 (1.03–3.35) | 2.94 (1.3–6.69) | 4.18 (1.63–10.7) | | 6.55 (2.46–17.5) |
| 10–15 years | 3.9 (1.79–8.5) | 1.88 (1.02–3.46) | 3.63 (1.6–8.25) | 3.67 (1.4–9.58) | | 6.49 (2.39–17.6) |
| 15–20 years | 4.06 (1.82–9.03) | 1.82 (0.97–3.42) | 3.1 (1.31–7.32) | 3.67 (1.38–9.78) | | 5.93 (2.18–16.1) |
| >20 years | 2.51 (1.13–5.58) | 1.79 (0.95–3.35) | 1.86 (0.8–4.31) | 2.44 (0.91–6.49) | | 4.48 (1.65–12.2) |
| Respondent ethnicity (ref. is White) | | | | | | |
| Black/African- American | 0.58 (0.42–0.81) | 0.58 (0.42–0.79) | 0.38 (0.25–0.55) | 0.63 (0.43–0.93) | | 0.45 (0.31–0.66) |
| Asian/Pacific Islander | 0.45 (0.33–0.63) | 0.5 (0.36–0.7) | 0.37 (0.26–0.54) | 0.55 (0.38–0.8) | | 0.37 (0.25–0.55) |
| Hispanic | 0.55 (0.38–0.79) | 0.69 (0.5–0.96) | 0.55 (0.37–0.81) | 0.93 (0.64–1.35) | | 0.63 (0.43–0.93) |
| Other | 1.01 (0.74–1.38) | 1.13 (0.84–1.51) | 0.91 (0.64–1.3) | 1.01 (0.71–1.43) | | 0.88 (0.62–1.25) |
| Respondent age (ref is ≥60) | | | | | | |
| <20–29 | 0.84 (0.5–1.43) | 0.93 (0.57–1.52) | 0.73 (0.4–1.31) | 0.98 (0.53–1.8) | | 1.39 (0.74–2.61) |
| 30–39 | 1.25 (0.91–1.7) | 1.17 (0.86–1.6) | 0.8 (0.55–1.16) | 0.91 (0.63–1.32) | | 1.35 (0.95–1.92) |
| 40–49 | 1.23 (0.94–1.62) | 1.09 (0.84–1.43) | 0.84 (0.61–1.14) | 1.19 (0.87–1.62) | | 1.15 (0.84–1.57) |
| 50–59 | 1.05 (0.82–1.35) | 1.06 (0.83–1.36) | 0.86 (0.64–1.15) | 0.99 (0.74–1.32) | | 1.14 (0.85–1.52) |
| Female sex (versus male) | 0.76 (0.62–0.92) | 0.68 (0.57–0.82) | 0.7 (0.56–0.87) | 0.73 (0.58–0.9) | | 0.6 (0.48–0.75) |
| Role in primary care (ref. is provider) | | | | | | |
| Administrative | 0.42 (0.31–0.58) | 0.72 (0.54–0.96) | 0.31 (0.21–0.44) | 0.61 (0.42–0.89) | | 0.35 (0.24–0.51) |
| Dietician | 0.19 (0.04–0.84) | 0.56 (0.21–1.49) | 0.11 (0.02–0.54) | 0.14 (0.02–1.03) | | 0.05 (0.01–0.41) |
| LPN/LVN/CNA | 0.54 (0.41–0.71) | 0.73 (0.57–0.93) | 0.45 (0.33–0.61) | 0.48 (0.35–0.65) | | 0.47 (0.34–0.64) |
| Mental Health Prof. | 0.71 (0.34–1.49) | 0.88 (0.44–1.75) | 0.39 (0.15–1.05) | 0.88 (0.35–2.2) | | 0.35 (0.13–0.92) |
| Nurse Care Manager | 0.68 (0.53–0.86) | 0.88 (0.69–1.12) | 0.59 (0.44–0.79) | 0.63 (0.48–0.82) | | 0.55 (0.42–0.73) |
| Nurse Case Manager | 0.61 (0.41–0.92) | 0.83 (0.55–1.25) | 0.49 (0.3–0.8) | 0.67 (0.42–1.07) | | 0.44 (0.27–0.73) |
| Other | 0.26 (0.1–0.72) | 0.74 (0.33–1.68) | 0.49 (0.21–1.13) | 0.37 (0.14–1.01) | | 0.31 (0.13–0.73) |
| Other RN | 0.51 (0.34–0.76) | 0.84 (0.57–1.23) | 0.49 (0.32–0.75) | 0.64 (0.41–1) | | 0.48 (0.31–0.74) |
| Pharmacist | 0.39 (0.24–0.63) | 0.59 (0.37–0.94) | 0.41 (0.23–0.7) | 0.3 (0.17–0.53) | | 0.22 (0.12–0.4) |
| Social Worker | 0.5 (0.25–1) | 0.44 (0.22–0.9) | 0.36 (0.16–0.78) | 0.41 (0.15–1.11) | | 0.2 (0.08–0.47) |
| Technician | 0.51 (0.31–0.84) | 0.57 (0.37–0.88) | 0.39 (0.23–0.64) | 0.99 (0.59–1.65) | | 0.46 (0.28–0.77) |
| PACT team member (ref. is Yes) | | | | | | |
| No | 0.72 (0.48–1.08) | 0.74 (0.51–1.07) | 0.74 (0.48–1.14) | 0.81 (0.52–1.27) | | 0.63 (0.4–0.98) |
| Not in Teamlet | 0.79 (0.57–1.09) | 0.76 (0.56–1.04) | 1.03 (0.73–1.46) | 0.9 (0.61–1.32) | | 0.78 (0.55–1.11) |
| Not sure | 3.78 (1.97–7.24) | 2.59 (1.4–4.76) | 3.03 (1.31–7.03) | 2.53 (1.25–5.16) | | 3.06 (1.3–7.24) |
| Time in primary care (ref. is >80%) | | | | | | |
| <20% | 0.59 (0.37–0.95) | 0.7 (0.47–1.06) | 0.52 (0.32–0.84) | 0.92 (0.55–1.54) | | 0.83 (0.51–1.35) |
| 20%–40% | 0.54 (0.3–1) | 0.79 (0.47–1.35) | 0.89 (0.5–1.57) | 1.04 (0.59–1.84) | | 0.88 (0.5–1.55) |
| 41%–60% | 0.86 (0.53–1.4) | 0.89 (0.57–1.39) | 0.78 (0.47–1.3) | 0.83 (0.48–1.43) | | 0.7 (0.41–1.22) |
| 61%–80% | 0.92 (0.63–1.36) | 1.09 (0.76–1.58) | 0.61 (0.38–0.97) | 0.7 (0.43–1.15) | | 0.91 (0.58–1.43) |
| Facility complexity (ref. is “1” or “most complex”) | | | | |  |  |
| 2 | 1.22 (0.9–1.67) | 1.15 (0.86–1.54) | 1.12 (0.82–1.52) | 1.09 (0.8–1.49) | | 1.09 (0.82–1.46) |
| 3 | 1.08 (0.78–1.51) | 1.06 (0.78–1.45) | 1.09 (0.79–1.52) | 0.99 (0.71–1.38) | | 1.02 (0.75–1.39) |
